# Environmental Stability and Infectivity of Hepatitis C Virus (HCV) in Different Human Body Fluids

**DOI:** 10.3389/fmicb.2018.00504

**Published:** 2018-03-27

**Authors:** Stephanie Pfaender, Fabian A. Helfritz, Anindya Siddharta, Daniel Todt, Patrick Behrendt, Julia Heyden, Nina Riebesehl, Wiebke Willmann, Joerg Steinmann, Jan Münch, Sandra Ciesek, Eike Steinmann

**Affiliations:** ^1^Institute of Virology and Immunology, Mittelhäusern, Switzerland; ^2^Department of Infectious Diseases and Pathobiology, Vetsuisse Faculty, University of Bern, Bern, Switzerland; ^3^Department of General, Visceral and Transplantation Surgery, University Hospital of Essen, University of Duisburg-Essen, Essen, Germany; ^4^Institute of Experimental Virology, TWINCORE-Centre for Experimental and Clinical Infections Research, a joint venture between the Hannover Medical School and the Helmholtz Centre for Infection Research, Hanover, Germany; ^5^Department of Molecular and Medical Virology, Ruhr-University Bochum, Bochum, Germany; ^6^Department of Gastroenterology, Hepatology and Endocrinology, Hannover Medical School, Hanover, Germany; ^7^Institute of Medical Microbiology, University Hospital of Essen, University of Duisburg-Essen, Essen, Germany; ^8^Institute for Clinical Hygiene, Medical Microbiology and Clinical Infectiology, Paracelsus Medical Private University, Nürnberg Hospital, Nürnberg, Germany; ^9^Institute of Molecular Virology, Ulm University Medical Center, Ulm, Germany; ^10^Institute for Virology, University Hospital of Essen, University of Duisburg-Essen, Essen, Germany

**Keywords:** hepatitis C virus, infectivity, saliva, semen, tear, contact lens solution, cerebrospinal fluid

## Abstract

**Background:** Hepatitis C virus (HCV) is a hepatotropic, blood-borne virus, but in up to one-third of infections of the transmission route remained unidentified. Viral genome copies of HCV have been identified in several body fluids, however, non-parental transmission upon exposure to contaminated body fluids seems to be rare. Several body fluids, e.g., tears and saliva, are renowned for their antimicrobial and antiviral properties, nevertheless, HCV stability has never been systematically analyzed in those fluids.

**Methods:** We used state of the art infectious HCV cell culture techniques to investigate the stability of HCV in different body fluids to estimate the potential risk of transmission via patient body fluid material. In addition, we mimicked a potential contamination of HCV in tear fluid and analyzed which impact commercially available contact lens solutions might have in such a scenario.

**Results:** We could demonstrate that HCV remains infectious over several days in body fluids like tears, saliva, semen, and cerebrospinal fluid. Only hydrogen-peroxide contact lens solutions were able to efficiently inactivate HCV in a suspension test.

**Conclusion:** These results indicate that HCV, once it is present in various body fluids of infected patients, remains infective and could potentially contribute to transmission upon direct contact.

## Introduction

Chronic HCV infection affects more than 71 million people worldwide and can lead to liver diseases like chronic hepatitis, cirrhosis, and hepatocellular carcinoma (Manns et al., 2017). One hallmark of HCV is its high degree of sequence variability which likely contributes to its ability to establish chronic infections. Different patient isolates are grouped into seven genotypes and more than 100 subtypes within the genus *Hepaciviridae* of the family *Flaviviridae* (Smith et al., 2014). While several potent DAAs have reached the market in the recent years, there is still no vaccine against HCV available (Tarr et al., 2015). Therefore, health care workers and other individuals like PWIDs or dialysis patients are at constant risk to acquire an HCV infection due to cross-transmission and accidental or occupational exposure. While it is well established that direct blood contact with infected individuals may cause an acute HCV infection, other transmission routes are uncertain and in up to one-third of HCV infected patients the cause of infection remained unclear (Ponde, 2011).

Several mechanical, chemical, and/or biological barriers protect humans from viral or bacterial infection. For example, the flushing action of tears and urine mechanically expels pathogens, while enzymes such as lysozyme and phospholipase A2 in saliva, tears, and breast milk can exert antimicrobial properties (Stiehm and Keller, 2001; Malamud et al., 2011; McDermott, 2013). Several studies have reported that HCV RNA can be detected in different human body fluids of HCV-infected patients such as saliva, semen, urine, sweat, and tears (Numata et al., 1993; Mendel et al., 1997; Leruez-Ville et al., 2000; Bourlet et al., 2002; Ortiz-Movilla et al., 2002; Suzuki et al., 2005; Wang et al., 2006). However, only serum has been demonstrated to be infectious in humans or experimental animal models (Billerbeck et al., 2013; Pfaender et al., 2016) and the infection risk associated with various human body fluids is not well defined. A reason for this ambiguity in transmission risks is that infection experiments with primary HCV material are technically not possible and cell culture assays permissive to HCV infection have not been available for a long time (Steinmann and Pietschmann, 2013). This obstacle has been overcome with the development of an HCV cell culture system based on the JFH HCV isolate (Wakita et al., 2005) and in fact different studies have recently assessed the stability of HCV and its susceptibility to different biocides using this system (Ciesek et al., 2010; Doerrbecker et al., 2011, 2013). Further, we could recently show that human breast milk exerts an antiviral effect against HCV due to endogenous lipase-dependent generation of free fatty acids, which destroys the viral lipid envelope of HCV (Pfaender et al., 2013). However, the anti- or pro-viral effects of other human body fluids have not been investigated so far.

In this study, we investigated the environmental stability of HCV in saliva, semen, CSF, and tears, the later in combination with contact lens solutions, to elucidate potential transmission risks or inactivation properties of these human body fluids.

## Materials and Methods

### Cell Culture and Reagents

For HCV infection experiments a human hepatoma cell line, designated Huh7.5, was used which is permissive for HCV infection and replication (Steinmann et al., 2008). The cells were grown in Dulbecco’s Modified Eagle Medium (DMEM; Invitrogen, Karlsruhe, Germany) supplemented with 2 mM L-glutamine, non-essential amino acids, 100 U of penicillin per mL, 100 μg of streptomycin per mL, and 10% fetal calf serum (DMEM complete).

### Plasmids and *in Vitro* Transcription

The plasmid pFK-Jc1 has been described recently and encodes the intragenotypic 2a/2a chimeric virus Jc1 (Pietschmann et al., 2006). *In vitro* transcripts of the individual constructs were generated by linearizing 5–10 μg of the Jc1 plasmid by digestion for 1 h with *Mlu* I. Plasmid DNA was extracted with phenol and chloroform and after precipitation with ethanol dissolved in RNase-free water. *In vitro* transcription reaction mixtures contained 80 mM HEPES (pH 7.5), 12 mM MgCl_2_, 2 mM spermidine, 40 mM dithiothreitol (DTT), a 3.125 mM concentration of each ribonucleoside triphosphate, 1 U of RNasin (Promega, Mannheim, Germany) per μL, 0.1 μg plasmid DNA/μL and 0.6 U of T7 RNA polymerase (Promega) per μL. After incubation for 2 h at 37°C, an additional 0.3 U of T7 RNA polymerase/μL reaction mixture was added, followed by another 2 h at 37°C. Transcription was terminated by the addition of 1.2 U of RNase-free DNase (Promega) per μg of plasmid DNA and 30 min incubation at 37°C. The RNA was extracted with acidic phenol and chloroform, precipitated with isopropanol and dissolved in RNase-free water. The concentration was determined by measurement of the optical density at 260 nm. Denaturing agarose gel electrophoresis was used to check RNA integrity.

### Electroporation of Viral RNA and Production of Cell Culture-Derived HCV

For electroporation of HCV RNA into Huh7.5 cells, single-cell suspensions were prepared by trypsinization of monolayers and subsequent resuspension with DMEM complete. Huh7.5 cells were washed with phosphate-buffered saline (PBS), counted and resuspended at 1.5 × 10^7^ cells per mL in cytomix containing 2 mM ATP and 5 mM glutathione. Unless otherwise stated, 10 μg of *in vitro* transcribed RNA was mixed with 400 μL cell suspension by pipetting and then electroporated with a Gene Pulser system (Bio-Rad, Munich, Germany) in a cuvette with a gap width of 0.4 cm (Bio-Rad) at 975 μF and 270 V. Cells were immediately transferred to 16 mL complete DMEM and 8 mL of the cell suspension was seeded per well. Virus-containing culture fluids were harvested after 48, 72, and 96 h and concentrated using centricons (Centricon plus-70, Millipore, United States). For determination of viral infectivity cell-free supernatants were used to infect naive Huh7.5 target cells.

### Immunohistochemical Staining and Virus Titration

Virus titers were determined as described elsewhere (Ciesek et al., 2011). In brief, Huh7.5 cells were seeded in 96-well plates at a density of 1 × 10^4^ cells per well 24 h prior to inoculation with dilutions of filtered cell culture supernatant (at least six wells were used per dilution). Two to three days later, cells were washed with PBS, fixed for 20 min with ice-cold methanol at -20°C, washed three times with PBS and then permeabilized and blocked for 1 h with PBS containing 0.5% saponin, 1% bovine serum albumin, 0.2% dried skim milk, and 0.02% sodium acid. Endogenous peroxidases were blocked by incubating cells for 5 min with PBS containing 0.3% hydrogen peroxide. After three times washing with PBS and once with PBS containing 0.5% saponin (PBS – saponin), NS5A was detected with a 1:1,000 dilution of hybridoma supernatant 9E10 in PBS – saponin for 1 h at RT or overnight at 4°C. Cells were washed as described above, and bound 9E10 antibody was detected by incubation with peroxidase – conjugated antibodies specific to murine IgG (Sigma-Aldrich, Steinheim, Germany) diluted 1:200 in PBS – saponin. After 1 h incubation at RT cells were washed as specified above. Finally, peroxidase activity was detected by using the Vector NovaRED substrate kit (Linaris, Wertheim, Germany) and TCID_50_ was determined.

### Testing of HCV Stability in Different Body Liquids

To test if HCV stability changes in different body liquids, virus diluted in tears (1:8), CSF (1:9), semen (1:9), or saliva (1:9) were incubated at room temperature for the respective time period. Effect of body liquid on HCV stability was compared to a virus suspension containing the respective amount of PBS. After the incubation period, target cells were infected in a limiting dilution assay on Huh7.5 cells. The TCID_50_ was determined 72 h post infection as described before (Steinmann et al., 2008).

### Virucidal Activity of Contact Lens Solutions

The virucidal activity testing of eight different contact lens solutions (**Table 1**) was carried out by mixing two part of test virus suspension with eight parts of the respective contact lens solutions in analogy to a virus suspension test described before (Ciesek et al., 2010). After different incubation times, the test mixtures were immediately serially diluted in DMEM and virus titers determined by TCID_50_. To analyze the effect of tear fluids on the virucidal activity of contact lens solutions, one part of test virus suspension, one part tear fluid and eight parts of contact lens solutions were mixed and tested as described above.

**Table 1:** Contact lens solutions employed in this study and their active ingredients.

Trade name	Manufacturer	Active ingredients
AOSept^®^ Plus	CIBA Vision	3.0% hydrogen peroxide
OPTI-FREE^®^ ever moist	Alcon	0.001% polyquaternium-1 0.0006% myristamidopropyl dimethylamine
iWear All-in-1 Basic	Sauflon	–
Boston Simplus	Bausch and Lomb	Poloxamine
COMPLETE^®^ Multi-Purpose Solution	Abbott	0.0001% polyhexamethylene biguanide 0.05% poloxamer 237 0.02% edetate disodium
Total Care	Abbott	0.0005% polyhexamethylene biguanide 0.01% edetate disodium
Best View All-in-One	ROSSMANN	0.0001% polyhexamethylene biguanide 0.01% edetate disodium
Oculsoft^®^ comfort	Bausch and Lomb	0.0005% polyaminopropyl biguanide 0.003% chlorhexidine gluconate 0.05% edetate disodium


### Statistics

For statistical analyses either one-way ANOVA followed by Dunnett’s multiple comparisons test or two-way ANOVA followed by Sidak’s multiple comparisons test was performed using GraphPad Prism version 7.03 for Windows, GraphPad Software, La Jolla, CA, United States, www.graphpad.com.

### Ethical Aspects

All body fluids were obtained from anti-HCV and HCV RNA negative healthy volunteers. The CSF samples were used after performing a conventional microbiological diagnosis. The study did not include patient’s details and did not result in additional constraints for the patients. All data were anonymously analyzed. The study had been approved by the ethics committee of Hannover Medical School, Germany.

## Results

### Infectious HCV Can Persist in Saliva for a Prolonged Time

**FIGURE 1:**
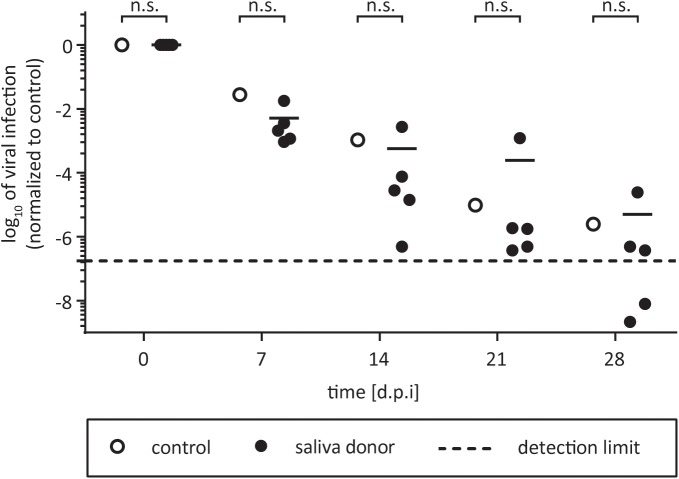
Stability of HCV in human saliva. One part of virus was added to one part of saliva and incubated for 7, 14, 21, or 28 days at room temperature. The saliva samples of five individual donors were used (black circles). Saliva was filtered (0.45 μm) and stored at 4°C before testing. In the untreated control the saliva was replaced with PBS (open circles). After incubation, mixtures were stored at –80°C until viral titers were determined by a limiting dilution assay to calculate the tissue culture infection dose (TCID_50_/mL). Data were normalized to the infection at time point zero of each experiment. Dashed line indicates assay background, n.s., not significant.

Saliva is well described for its antimicrobial effects due to different enzymes (Malamud et al., 2011). Nevertheless, several human viruses can be efficiently transmitted by saliva by direct transfer from infected individuals to non-infected persons (Ross, 1971). HCV RNA has been detected in saliva of infected patients (Suzuki et al., 2005; Wang et al., 2006), however, its infective potential has not been analyzed so far. To evaluate HCV stability in saliva and the influence of potential defense mechanisms present within this body fluid, HCV and saliva were incubated in suspension for selected time points up to 28 days at room temperature. As control, virus was incubated with PBS. To take donor variations into account, saliva samples from five individual donors were used. Infectivity was determined by limiting dilution assay. HCV infectivity was detectable up to 21 days at room temperature in all samples, with minor difference between saliva and control incubated samples (**Figure 1**). For one donor, even higher viral titers could be observed compared to the control incubation. The overall infectivity decreased in a time-dependent manner, however, no antiviral effect of saliva on HCV infectivity could be observed. Four weeks post inoculation, HCV infectivity dropped below the detection limit in only two out of five donor samples (**Figure 1**). In conclusion, HCV is stable in saliva for up to 3 weeks at room temperature and remains its infective potential.

### Semen Does Not Enhance or Abrogate HCV Infectivity

**FIGURE 2:**
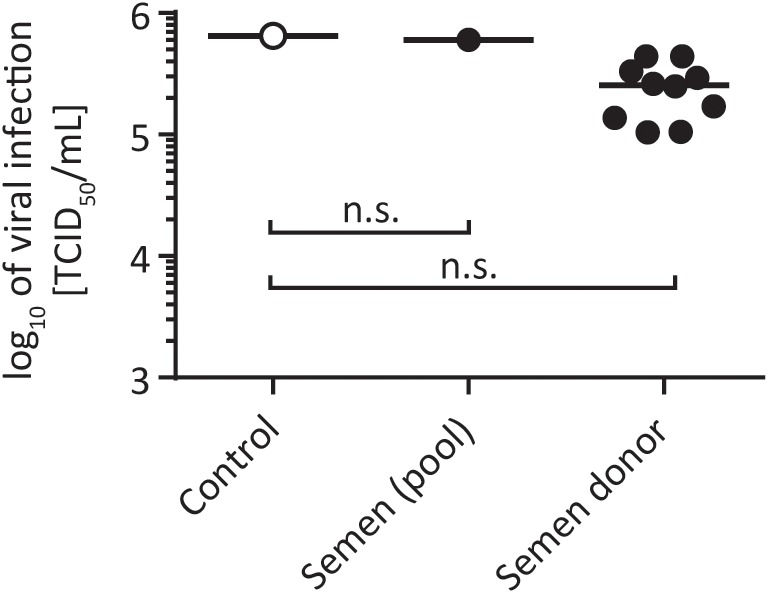
Stability of HCV in human semen. One part of virus was added to nine parts of semen and incubated for 24 h at room temperature. A pool of 30 different semen samples and semen samples from nine individual donors were used (black circles). In the untreated control the sperm was replaced with PBS (open circles). Viral titers were determined by a limiting dilution assay to calculate the tissue culture infection dose (TCID_50_/mL), n.s., not significant.

Hepatitis C virus can be transmitted through sexual intercourse. Terrault et al. (2013) found that the maximum incidence rate of HCV transmission by sex was 0.07% per year, which is lower compared to other sexually transmitted diseases like HIV or herpes simplex virus type 2 (HSV-2). Especially for HIV, it has been described that semen enhances HIV infectivity (Münch et al., 2007), thereby facilitating viral transmission. We next investigated the influence of semen on HCV infectivity. To this end, we collected semen from healthy volunteers and incubated it together with HCV in suspension for 24 h at room temperature. Infectivity was determined by a limiting dilution assay after 72 h. In total, a pool of 30 different semen samples, encompassing semen samples from 10 individual donors were used. As shown in **Figure 2**, none of the tested semen samples enhanced or abrogated HCV infectivity under these experimental conditions (**Figure 2**). These results indicate that potential infectious virus present in semen remains stable and infectivity seems not to be influenced by seminal fluid.

### Cerebrospinal Fluid Has No Pro- or Antiviral Effects on HCV

**FIGURE 3:**
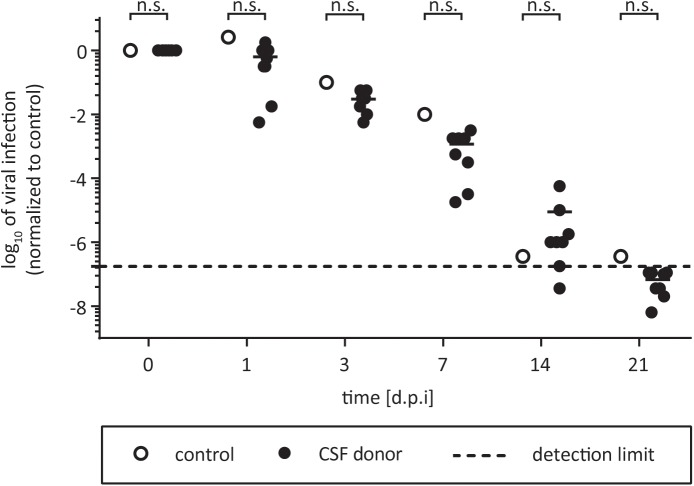
Effect of CSF on HCV. One part of virus was added to nine parts of CSF and incubated for 7, 14, 21, or 28 days at room temperature. The CSF samples from eight individual donors were used (black circles). In the untreated control the liquor is replaced with PBS (open circles). Samples were stored at –80°C until viral titers were determined by a limiting dilution assay to calculate the tissue culture infection dose (TCID_50_/mL). Data were normalized to the infection at time point zero of each experiment. Dashed line indicates assay background, n.s., not significant.

Even though HCV exhibits a liver tropism, extrahepatic manifestations have been described (Negro et al., 2015). It is still a long debate if HCV infects the brain, however, up to 20–80% of patients with chronic HCV exhibit symptoms like chronic fatigue (Yarlott et al., 2017). In addition, there are several reports of deficits in attention, concentration, and memory or depression (Yarlott et al., 2017). Furthermore, HCV RNA has been detected in CSF and the brain of chronically infected patients with neuropathological abnormalities (Maggi et al., 1999; Laskus et al., 2002; Vargas et al., 2002). Fletcher et al. (2012) have shown that brain microvascular endothelial cells (BMECs), are permissive to HCV infection in culture, however, if CSF itself might have pro- or antiviral effects is unknown. To address this question, we tested HCV infectivity in CSF of eight different individuals by incubation for selected time points at room temperature. As control, virus was incubated with PBS in suspension. We monitored viral stability in CSF for up to 21 days, and could not detect any pro- or antiviral effects on HCV (**Figure 3**). The virus remained infectious upon the time course. This indicates that virus which potentially crosses the blood–brain barrier and enters the CSF retains its infectious capacity for several weeks.

### Tears Do Not Decrease HCV Infectivity but Can Abrogate the Antiviral Effects of a Contact Lens Solution

**FIGURE 4:**
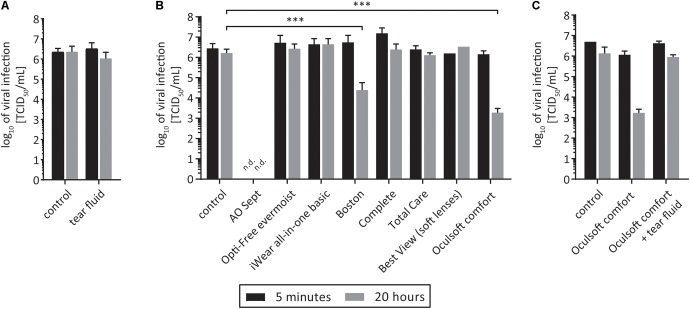
Effect of tear fluid and contact lens cleaning solutions on HCV. **(A)** Eight parts of medium were incubated with one part of tear fluid and on part of virus suspension for 5 min (black bars) or 20 h (gray bars) at room temperature. For the untreated control PBS was used. Viral titers were determined by a limiting dilution assay to calculate the tissue culture infection dose (TCID_50_/mL). **(B)** One part of virus was added to nine parts of the selected contact lens cleaning solution and incubated for 5 min (black bars) or 20 h (gray bars) at room temperature. For the untreated control PBS was used. Viral titers were determined by a limiting dilution assay to calculate the tissue culture infection dose (TCID_50_/mL). n.d., not detected above toxicity background, ^∗∗∗^*p* ≤ 0.001. **(C)** Eight parts of contact lens solution *Oculsoft comfort* were incubated with one part of tear fluid and on part of virus suspension for 5 min (black bars) or 20 h (gray bars). For the untreated control PBS was used. Tear fluid was replaced in the control with PBS. Viral titers were determined by a limiting dilution assay to calculate the tissue culture infection dose (TCID_50_/mL).

Tear fluid from chronically HCV infected patients has been shown to contain viral genome copies as determined via qRT-PCR (Feucht et al., 1994; Mendel et al., 1997; Komatsu et al., 2012). However, there are no indications that HCV can be transmitted via tears. It is known, that tear fluid contains several antiviral and antimicrobial components (Chandler and Gillette, 1983). To investigate if tear fluid can potentially inactivate HCV, tear fluid of a healthy volunteer and virus suspension were incubated for selected time points at room temperature. As shown in **Figure 4A**, we were not able to detect any effect on HCV infectivity 5 min and 20 h after incubation with tears indicating that potential antimicrobial factors present in tear fluid are not able to inactivate HCV sufficiently (**Figure 4A**).

Given the well documented presence of HCV RNA in tears, we next wanted to evaluate the effects of contact lens solutions on viral stability. To this end, we chose eight different commercially available contact lens solutions for cleaning and storage of contact lenses (**Table 1**). The contact lens solutions were based on different ingredients like hydrogen peroxide, polyquaternium, myristamidopropyl dimethylamine, and polyhexanide. To analyze viral stability, one part of HCV was added to nine parts of the selected contact lens cleaning solution and incubated for designated time points at room temperature. Only one solution (AOSept^®^ Plus; “AO sept”), which is based on hydrogen peroxide was able to inactivate HCV infectivity quite rapidly and completely, whereas the other solutions demonstrated low antiviral activity or were able to reduce viral titers only after a prolonged incubation time of 20 h (**Figure 4B**). We chose one contact lens solution, namely “Oculsoft comfort,” which was able to efficiently reduce viral titers after a prolonged incubation period of 20 h, to mimic a more physiological situation, and to investigate if the antiviral effect was influenced due to the presence of tear fluid. Therefore, we incubated for 5 min and 20 h solution “Oculsoft comfort” with tear fluid and virus suspension and determined viral titers by a limiting dilution assay. Interestingly, in the presence of tear fluid the inhibitory effect on HCV infectivity of the contact lens solution was abrogated (**Figure 4C**). In conclusion, we could show that HCV infectivity remains stable in tear fluid and that commercially available contact lens solutions are rarely sufficient to inactivate a potentially contaminated lens.

## Discussion

Several viruses are known to be transmitted via saliva, with the classical example being Epstein–Barr virus-induced mononucleosis, which is broadly known as kissing disease (Niederman et al., 1970). There are several studies that describe the presence of HCV RNA in the saliva of about 39–72% of chronically infected patients (Ferreiro et al., 2005). However, most epidemiological studies suggest that HCV transmission via contaminated saliva is low and there is no evidence that HCV can easily be spread via salivary exchange, e.g., upon kissing, sneezing, coughing, etc. (Ferreiro et al., 2005). There seems to be a correlation between the presence of virus in saliva and the viral load in blood, however, viral levels in saliva are in general low compared to blood levels (Wang et al., 1992; Numata et al., 1993). It is not known whether there are specific defense mechanisms, e.g., due to the presence of neutralizing antibodies or non-specific defense mechanisms, like antiviral enzymes, that contribute to this observed low transmission route. To analyze potential antiviral mechanisms in the saliva of healthy individuals, we tested HCV stability over a prolonged time. HCV infectivity was surprisingly not altered when incubated with saliva and remained stable for up to 1 month. It is possible that the observed low level of virus present in saliva is not sufficient to mediate an efficient transmission; however, the potential risk should not be neglected, especially upon scenarios where the natural barrier has been disrupted.

While HIV is mainly transmitted via sexual intercourse, transmission risk for HCV via this route seems to be rather low and is associated with specific risk groups like MSM (Butler et al., 2010). While semen derived fibrils have been demonstrated to enhance HIV infectivity (Münch et al., 2007), the influence of seminal fluid on HCV stability is not clear. We therefore aimed to analyze viral infectivity in the semen from healthy donors. Unlike HIV, we could demonstrate that HCV infectivity was not affected by semen, as it neither abrogated nor enhanced viral infectivity, suggesting that seminal amyloid does also not affect HCV infectivity.

Several HCV patients experience significant changes in their physical and mental well-being, which are most commonly manifested as fatigue and depression. Indeed, the brain has been proposed as an extrahepatic replication site for HCV with endothelial cells from the blood–brain barrier and peripheral neuroblastoma cells being able to support HCV infection (Burgel et al., 2011; Fletcher et al., 2012). Laskus et al. (2002) were able to show that around 60% of all patients with chronic HCV had detectable HCV RNA levels in the CSF. A recent study even revealed genetic compartmentalization in the CSF from cogitative impaired patients supporting the theory for an extrahepatic manifestation (Tully et al., 2016). CSF contains >99% water, low amounts of plasma proteins and glucose and sporadic lymphocytes (Spector et al., 2015). Antimicrobial properties are not described, but the low amounts of proteins and glucose might lead to a reduced stability of viruses in CSF. We here show that HCV incubated in CSF is as stable as HCV incubated in PBS. We could confirm that CSF has no antiviral activity against HCV and that HCV can survive in CSF for a longer period of time.

Next to the other body fluids, HCV RNA has been readily detected in the tear fluid of HCV-infected patients (Feucht et al., 1994; Mendel et al., 1997). Also for other viruses like HIV, HBV, or Ebola Virus tears have been described as viral positive body fluid (Su et al., 1994; Han et al., 2011; Vetter et al., 2016). Importantly, HBV isolated from tears of chronically infected patients has even been shown to be infectious in the animal model (Komatsu et al., 2012), emphasizing a possible viral transmission risk via tear fluid. Nevertheless, tear-mediated transmission of HCV has not been described so far. Tears are known to contain a number of antimicrobial components which help to protect the eye from infection (Flanagan and Willcox, 2009). One prominent component is lactoferrin which has been described for its antimicrobial and anti-inflammatory properties (Flanagan and Willcox, 2009). To investigate whether HCV is able to retain its infectivity within tear fluid, we analyzed its stability upon artificial contamination with HCV and could observe no decrease in viral titer. This indicates that possible antiviral components described to be present in tear fluid, like lactoferrin, are not sufficient to inactivate HCV. Next, we tested additionally if commercially available contact lens solutions were able to inactive a potential HCV contamination. Surprisingly, solutions which are not based on hydrogen peroxide were not able to readily inactivate HCV. Only two other solutions were able to reduce viral titers by several orders of magnitude after 20 h incubation. Even more important, the antiviral effect of one contact lens solution became abrogated in the presence of tear fluid. A phenomenon that has been also observed for different bacteria (Hildebrandt et al., 2012). These results show for the first time that several body fluids do not exert antiviral activities against HCV. However, to determine if they could be a possible source of HCV transmission in the environment, *in vivo* experiments with, e.g., humanized mice would be necessary. HCV RNA has also been detected in human sweat of HCV-positive patients (Ortiz-Movilla et al., 2002) as well as urine (Numata et al., 1993), however, both body fluids were not included in the present study for further analysis.

In summary, we present the first systematic investigation of HCV stability and infectivity in different body fluids. Even though transmission via saliva, tears, semen, or other non-parental body fluids are believed to be rare, we could clearly demonstrate that virus remains infective within these fluids and could potentially become transmitted. It is likely that the reduced viral loads in the diverse body fluids, as compared to viral levels in blood, are responsible for the observed low transmission rates, however, transmission might still be possible and its risk should not be fully neglected. In conclusion, strict compliance to established hygienic guidelines should be mandatory to avoid further HCV infections.

## Author Contributions

SP and FH performed the experiments, analyzed the data, and wrote the first draft of the manuscript. AS, JH, NR, and WW performed the experiments. DT analyzed the data. PB, JS, JM, and SC gave scientific input. ES designed the research and wrote the manuscript.

## Conflict of Interest Statement

The authors declare that the research was conducted in the absence of any commercial or financial relationships that could be construed as a potential conflict of interest. The reviewer SG declared a past co-authorship with one of the authors ES to the handling Editor. The reviewer DP and handling Editor declared their shared affiliation.

## Abbreviations

CSF: cerebrospinal fluid
DAA: direct acting antiviral drugs
HBV: hepatitis B virus
HCV: hepatitis C virus
HIV: human immunodeficiency virus
JFH: Japanese fulminant hepatitis
MSM: men who have sex with men
PWIDs: people who inject drugs
qRT-PCR: quantitative real time-polymerase chain reaction
SD: standard deviation
TCID_50_: tissue culture infectious dose 50

## References

[B1] Billerbeck E. De Jong Y. Dorner M. De La Fuente C. Ploss A. (2013). Animal models for hepatitis C. *Curr. Top. Microbiol. Immunol.* 369 49–86. 10.1007/978-3-642-27340-7_3 23463197

[B2] Bourlet T. Levy R. Maertens A. Tardy J. C. Grattard F. Cordonier H. (2002). Detection and characterization of hepatitis C virus RNA in seminal plasma and spermatozoon fractions of semen from patients attempting medically assisted conception. *J. Clin. Microbiol.* 40 3252–3255. 10.1128/JCM.40.9.3252-3255.2002 12202561 PMC130669

[B3] Burgel B. Friesland M. Koch A. Manns M. P. Wedemeyer H. Weissenborn K. (2011). Hepatitis C virus enters human peripheral neuroblastoma cells - evidence for extra-hepatic cells sustaining hepatitis C virus penetration. *J. Viral Hepat.* 18 562–570. 10.1111/j.1365-2893.2010.01339.x 20579278

[B4] Butler D. M. Delport W. Kosakovsky Pond S. L. Lakdawala M. K. Cheng P. M. Little S. J. (2010). The origins of sexually transmitted HIV among men who have sex with men. *Sci. Transl. Med.* 2:18re1. 10.1126/scitranslmed.3000447 20371483 PMC2945226

[B5] Chandler J. W. Gillette T. E. (1983). Immunologic defense mechanisms of the ocular surface. *Ophthalmology* 90 585–591. 10.1016/S0161-6420(83)34510-3 6888852

[B6] Ciesek S. Friesland M. Steinmann J. Becker B. Wedemeyer H. Manns M. P. (2010). How stable is the hepatitis C virus (HCV)? Environmental stability of HCV and its susceptibility to chemical biocides. *J. Infect. Dis.* 201 1859–1866. 10.1086/652803 20441517

[B7] Ciesek S. Von Hahn T. Colpitts C. C. Schang L. M. Friesland M. Steinmann J. (2011). The green tea polyphenol, epigallocatechin-3-gallate, inhibits hepatitis C virus entry. *Hepatology* 54 1947–1955. 10.1002/hep.24610 21837753

[B8] Doerrbecker J. Friesland M. Ciesek S. Erichsen T. J. Mateu-Gelabert P. Steinmann J. (2011). Inactivation and survival of hepatitis C virus on inanimate surfaces. *J. Infect. Dis.* 204 1830–1838. 10.1093/infdis/jir535 22013220 PMC3247810

[B9] Doerrbecker J. Meuleman P. Kang J. Riebesehl N. Wilhelm C. Friesland M. (2013). Thermostability of seven hepatitis C virus genotypes in vitro and in vivo. *J. Viral Hepat.* 20 478–485. 10.1111/jvh.12055 23730841

[B10] Ferreiro M. C. Dios P. D. Scully C. (2005). Transmission of hepatitis C virus by saliva? *Oral Dis.* 11 230–235. 15984954 10.1111/j.1601-0825.2005.01076.x

[B11] Feucht H. H. Polywka S. Zollner B. Laufs R. (1994). Greater amount of HCV-RNA in tears compared to blood. *Microbiol. Immunol.* 38 157–158. 10.1111/j.1348-0421.1994.tb01758.x 8041303

[B12] Flanagan J. L. Willcox M. D. (2009). Role of lactoferrin in the tear film. *Biochimie* 91 35–43. 10.1016/j.biochi.2008.07.007 18718499

[B13] Fletcher N. F. Wilson G. K. Murray J. Hu K. Lewis A. Reynolds G. M. (2012). Hepatitis C virus infects the endothelial cells of the blood-brain barrier. *Gastroenterology* 142 634.e6–643.e6. 10.1053/j.gastro.2011.11.028 22138189 PMC3801216

[B14] Han Y. Wu N. Zhu W. Li Y. Zuo L. Ye J. (2011). Detection of HIV-1 viruses in tears of patients even under long-term HAART. *AIDS* 25 1925–1927. 10.1097/QAD.0b013e32834b3578 21811142

[B15] Hildebrandt C. Wagner D. Kohlmann T. Kramer A. (2012). In-vitro analysis of the microbicidal activity of 6 contact lens care solutions. *BMC Infect. Dis.* 12:241. 10.1186/1471-2334-12-241 23033880 PMC3519705

[B16] Komatsu H. Inui A. Sogo T. Tateno A. Shimokawa R. Fujisawa T. (2012). Tears from children with chronic hepatitis B virus (HBV) infection are infectious vehicles of HBV transmission: experimental transmission of HBV by tears, using mice with chimeric human livers. *J. Infect. Dis.* 206 478–485. 10.1093/infdis/jis293 22508939

[B17] Laskus T. Radkowski M. Bednarska A. Wilkinson J. Adair D. Nowicki M. (2002). Detection and analysis of hepatitis C virus sequences in cerebrospinal fluid. *J. Virol.* 76 10064–10068. 10.1128/JVI.76.19.10064-10068.2002 12208987 PMC136534

[B18] Leruez-Ville M. Kunstmann J. M. De Almeida M. Rouzioux C. Chaix M. L. (2000). Detection of hepatitis C virus in the semen of infected men. *Lancet* 356 42–43. 10.1016/S0140-6736(00)02435-1 10892766

[B19] Maggi F. Giorgi M. Fornai C. Morrica A. Vatteroni M. L. Pistello M. (1999). Detection and quasispecies analysis of hepatitis C virus in the cerebrospinal fluid of infected patients. *J. Neurovirol.* 5 319–323. 10.3109/13550289909015819 10414523

[B20] Malamud D. Abrams W. R. Barber C. A. Weissman D. Rehtanz M. Golub E. (2011). Antiviral activities in human saliva. *Adv. Dent. Res.* 23 34–37. 10.1177/0022034511399282 21441478 PMC3144043

[B21] Manns M. P. Buti M. Gane E. Pawlotsky J. M. Razavi H. Terrault N. (2017). Hepatitis C virus infection. *Nat. Rev. Dis. Primers* 3:17006. 10.1038/nrdp.2017.6 28252637

[B22] McDermott A. M. (2013). Antimicrobial compounds in tears. *Exp. Eye Res.* 117 53–61. 10.1016/j.exer.2013.07.014 23880529 PMC3844110

[B23] Mendel I. Muraine M. Riachi G. El Forzli F. Bertin C. Colin R. (1997). Detection and genotyping of the hepatitis C RNA in tear fluid from patients with chronic hepatitis C. *J. Med. Virol.* 51 231–233. 10.1002/(SICI)1096-9071(199703)51:3<231::AID-JMV15>3.0.CO;2-N 9139089

[B24] Münch J. Rucker E. Standker L. Adermann K. Goffinet C. Schindler M. (2007). Semen-derived amyloid fibrils drastically enhance HIV infection. *Cell* 131 1059–1071. 10.1016/j.cell.2007.10.014 18083097

[B25] Negro F. Forton D. Craxi A. Sulkowski M. S. Feld J. J. Manns M. P. (2015). Extrahepatic morbidity and mortality of chronic hepatitis C. *Gastroenterology* 149 1345–1360. 10.1053/j.gastro.2015.08.035 26319013

[B26] Niederman J. C. Evans A. S. Subrahmanyan L. Mccollum R. W. (1970). Prevalence, incidence and persistence of EB virus antibody in young adults. *N. Engl. J. Med.* 282 361–365. 10.1056/NEJM197002122820704 4312365

[B27] Numata N. Ohori H. Hayakawa Y. Saitoh Y. Tsunoda A. Kanno A. (1993). Demonstration of hepatitis C virus genome in saliva and urine of patients with type C hepatitis: usefulness of the single round polymerase chain reaction method for detection of the HCV genome. *J. Med. Virol.* 41 120–128. 10.1002/jmv.1890410207 8283173

[B28] Ortiz-Movilla N. Lazaro P. Rodriguez-Inigo E. Bartolome J. Longo I. Lecona M. (2002). Hepatitis C virus replicates in sweat glands and is released into sweat in patients with chronic hepatitis C. *J. Med. Virol.* 68 529–536. 10.1002/jmv.10238 12376961

[B29] Pfaender S. Heyden J. Friesland M. Ciesek S. Ejaz A. Steinmann J. (2013). Inactivation of hepatitis C virus infectivity by human breast milk. *J. Infect. Dis.* 208 1943–1952. 10.1093/infdis/jit519 24068703

[B30] Pfaender S. Von Hahn T. Steinmann J. Ciesek S. Steinmann E. (2016). Prevention strategies for blood-borne viruses-in the Era of vaccines, direct acting antivirals and antiretroviral therapy. *Rev. Med. Virol.* 26 330–339. 10.1002/rmv.1890 27185010 PMC5084801

[B31] Pietschmann T. Kaul A. Koutsoudakis G. Shavinskaya A. Kallis S. Steinmann E. (2006). Construction and characterization of infectious intragenotypic and intergenotypic hepatitis C virus chimeras. *Proc. Natl. Acad. Sci. U.S.A.* 103 7408–7413. 10.1073/pnas.0504877103 16651538 PMC1455439

[B32] Ponde R. A. (2011). Hidden hazards of HCV transmission. *Med. Microbiol. Immunol.* 200 7–11. 10.1007/s00430-010-0159-9 20461405

[B33] Ross P. W. (1971). Quantitative studies on the salivary flora. *J. Clin. Pathol.* 24 717–720. 10.1136/jcp.24.8.717 4399776 PMC477140

[B34] Smith D. B. Bukh J. Kuiken C. Muerhoff A. S. Rice C. M. Stapleton J. T. (2014). Expanded classification of hepatitis C virus into 7 genotypes and 67 subtypes: updated criteria and genotype assignment web resource. *Hepatology* 59 318–327. 10.1002/hep.26744 24115039 PMC4063340

[B35] Spector R. Robert Snodgrass S. Johanson C. E. (2015). A balanced view of the cerebrospinal fluid composition and functions: focus on adult humans. *Exp. Neurol.* 273 57–68. 10.1016/j.expneurol.2015.07.027 26247808

[B36] Steinmann E. Brohm C. Kallis S. Bartenschlager R. Pietschmann T. (2008). Efficient trans-encapsidation of hepatitis C virus RNAs into infectious virus-like particles. *J. Virol.* 82 7034–7046. 10.1128/JVI.00118-08 18480457 PMC2446957

[B37] Steinmann E. Pietschmann T. (2013). Cell culture systems for hepatitis C virus. *Curr. Top. Microbiol. Immunol.* 369 17–48. 10.1007/978-3-642-27340-7_2 23463196

[B38] Stiehm E. R. Keller M. A. (2001). Breast milk transmission of viral disease. *Adv. Nutr. Res.* 10 105–122. 11795036 10.1007/978-1-4615-0661-4_5

[B39] Su C. S. Bowden S. Fong L. P. Taylor H. R. (1994). Detection of hepatitis B virus DNA in tears by polymerase chain reaction. *Arch. Ophthalmol.* 112 621–625. 10.1001/archopht.1994.01090170065024 8185518

[B40] Suzuki T. Omata K. Satoh T. Miyasaka T. Arai C. Maeda M. (2005). Quantitative detection of hepatitis C virus (HCV) RNA in saliva and gingival crevicular fluid of HCV-infected patients. *J. Clin. Microbiol.* 43 4413–4417. 10.1128/JCM.43.9.4413-4417.2005 16145085 PMC1234063

[B41] Tarr A. W. Khera T. Hueging K. Sheldon J. Steinmann E. Pietschmann T. (2015). Genetic diversity underlying the envelope glycoproteins of hepatitis C virus: structural and functional consequences and the implications for vaccine design. *Viruses* 7 3995–4046. 10.3390/v7072809 26193307 PMC4517138

[B42] Terrault N. A. Dodge J. L. Murphy E. L. Tavis J. E. Kiss A. Levin T. R. (2013). Sexual transmission of hepatitis C virus among monogamous heterosexual couples: the HCV partners study. *Hepatology* 57 881–889. 10.1002/hep.26164 23175457 PMC4384338

[B43] Tully D. C. Hjerrild S. Leutscher P. D. Renvillard S. G. Ogilvie C. B. Bean D. J. (2016). Deep sequencing of hepatitis C virus reveals genetic compartmentalization in cerebrospinal fluid from cognitively impaired patients. *Liver Int.* 36 1418–1424. 10.1111/liv.13134 27045383 PMC5553127

[B44] Vargas H. E. Laskus T. Radkowski M. Wilkinson J. Balan V. Douglas D. D. (2002). Detection of hepatitis C virus sequences in brain tissue obtained in recurrent hepatitis C after liver transplantation. *Liver Transpl.* 8 1014–1019. 10.1053/jlts.2002.36393 12424714

[B45] Vetter P. Fischer W. A. II Schibler M. Jacobs M. Bausch D. G. Kaiser L. (2016). Ebola virus shedding and transmission: review of current evidence. *J. Infect. Dis.* 214 S177–S184. 10.1093/infdis/jiw254 27443613 PMC6283352

[B46] Wakita T. Pietschmann T. Kato T. Date T. Miyamoto M. Zhao Z. (2005). Production of infectious hepatitis C virus in tissue culture from a cloned viral genome. *Nat. Med.* 11 791–796. 10.1038/nm1268 15951748 PMC2918402

[B47] Wang C. C. Morishima C. Chung M. Engelberg R. Krantz E. Krows M. (2006). High serum hepatitis C virus (HCV) RNA load predicts the presence of HCV RNA in saliva from individuals with chronic and acute HCV infection. *J. Infect. Dis.* 193 672–676. 10.1086/499602 16453262

[B48] Wang J. T. Wang T. H. Sheu J. C. Lin J. T. Chen D. S. (1992). Hepatitis C virus RNA in saliva of patients with posttransfusion hepatitis and low efficiency of transmission among spouses. *J. Med. Virol.* 36 28–31. 10.1002/jmv.1890360106 1315367

[B49] Yarlott L. Heald E. Forton D. (2017). Hepatitis C virus infection, and neurological and psychiatric disorders - A review. *J. Adv. Res.* 8 139–148. 10.1016/j.jare.2016.09.005 28149649 PMC5272938

